# Generalizability of deep learning–based dose conversion model in proton beam therapy

**DOI:** 10.1002/acm2.70528

**Published:** 2026-02-27

**Authors:** Ryohei Kato, Noriyuki Kadoya, Takahiro Kato, Ryota Tozuka, Shuta Ogawa, Masao Murakami, Keiichi Jingu

**Affiliations:** ^1^ Department of Radiation Physics and Technology Southern Tohoku Proton Therapy Center Koriyama Fukushima Japan; ^2^ Department of Radiation Oncology Tohoku University Graduate School of Medicine Sendai Miyagi Japan; ^3^ Department of Radiological Sciences School of Health Sciences Fukushima Medical University Fukushima Fukushima Japan; ^4^ Department of Radiology University of Yamanashi Chuo‐city Yamanashi Japan; ^5^ Department of Radiation Oncology Southern Tohoku Proton Therapy Center Koriyama Fukushima Japan

**Keywords:** deep learning, Monte Carlo, proton therapy

## Abstract

**Background:**

In proton beam therapy (PBT), the analytical pencil beam (PB) algorithm involves dose uncertainties in inhomogeneous regions, making accurate Monte Carlo (MC) dose calculation desirable but time‐consuming. Deep learning, converting the dose calculated by the PB algorithm into an MC‐equivalent dose distribution, can resolve the trade‐off between calculation accuracy and speed. Training a DL‐based dose conversion model that can be applied to any tumor site would be ideal; however, the appropriate training regions and its generalizability remain unclear.

**Purpose:**

We developed a DL‐based dose conversion model trained on four representative tumor sites (i.e., head and neck, lung, liver, and prostate), and evaluated its generalizability.

**Methods:**

Data from 339 patients (a total of 1147 beams) were used. PB doses were obtained from the treatment planning system, and MC doses were calculated using an in‐house MC platform. Our developed DL‐based dose conversion model was designed to input a treatment planning computed tomography image and PB dose in a single field and output an MC‐equivalent dose. The model's generalizability was evaluated on untrained tumor sites, including the esophagus, pancreas, colorectum, brain, breast, cervix, and limb bone and soft tissue. The conversion performance was assessed using 3D γ‐analysis and the Dice similarity coefficient (DSC) for isodose volumes.

**Results:**

For most untrained tumor sites, the model achieved average γ‐passing rates of ≥90% with a criterion of 3%/2 mm. The esophagus, breast, which are close to the lung, and limb bone and soft tissue showed slightly lower passing rates of 91.3%, 85.9%, and 89.1%, respectively. The average DSC values exceeded 0.8 for most untrained tumor sites.

**Conclusion:**

The proposed DL‐based dose conversion model demonstrated high accuracy and generalizability, even for untrained tumor sites. These findings suggest that the model can be adapted to biases in collecting disease data at each PBT center and for rare diseases.

## 1 INTRODUCTION

Highly accurate dose calculations are crucial in radiotherapy to deliver the optimal dose distribution to the patient. In proton beam therapy (PBT), the analytical pencil beam (PB) algorithm and Monte Carlo (MC) dose calculation are primarily used in clinical settings.^1^ The PB algorithm utilizes measured depth dose distributions and lateral scattering parameters to calculate the dose distribution within a patient's body for each PB.^2^ The PB algorithm can calculate dose distributions rapidly; however, its accuracy decreases in heterogeneous regions, for example, the lungs and head and neck (H&N).^3^, ^4^, ^5^, ^6^, ^7^, ^8^, ^9^, ^10^ This reduction in accuracy leads to inaccurate estimations of the range and Bragg peak within the heterogeneous patient's body, thereby affecting the quality of PBT. Recently, the highly accurate MC dose calculation has been implemented in some proton treatment planning systems (TPSs) and is primarily used for pencil beam scanning (PBS) systems.^1^, ^7^, ^8^, ^10^ Conventionally, the MC dose calculation has been problematic because of its long computational time; however, by utilizing a graphics processing unit (GPU) and limiting physical interactions, it is possible to reduce the calculation time while maintaining clinically acceptable accuracy.^11^, ^12^, ^13^ Nevertheless, the MC dose calculation cannot be used at centers that do not have a TPS equipped with the MC dose calculation or where the MC dose calculation in the TPS is incompatible with PBT machines, for example, passive scattering systems. In such PBT centers, the PB algorithm is still used clinically.^1^

In recent years, deep learning (DL) methods have attracted increasing attention in radiotherapy, and DL models that convert dose distributions calculated using low‐accuracy algorithms into doses calculated using high‐accuracy algorithms have been proposed.^14^, ^15^ A DL model that converts PB doses to MC doses has also been developed for PBT.^16^ The DL‐based dose conversion model can instantly calculate highly accurate dose distributions equivalent to the MC dose calculation, even at centers where MC methods cannot be employed in TPS, potentially improving the accuracy of treatment planning. In addition, the DL‐based dose conversion method is expected to be applied to online adaptive PBT in the future by utilizing the high‐speed dose calculations of the PB algorithm.

Generally, a large amount of training data is required to improve the performance of DL models. The DL‐based dose prediction in PBT has been reported for the H&N,^17^, ^18^ lung,^19^ and prostate.^18^, ^19^, ^20^ Typically, dose prediction models train each tumor site because the treatment planning concept and protocol vary for each tumor site and patient. Furthermore, the DL‐based dose conversion model can calculate MC‐equivalent doses by interpolating the PB algorithm uncertainties in inhomogeneous regions, irrespective of the tumor site or treatment planning concept. Therefore, training various tumor sites with different inhomogeneities is expected to improve model performance. However, the variation and number of diseases that can be collected at each PBT center are limited; therefore, collecting a substantial amount of treatment planning data for all tumor sites is challenging. In addition, collecting data on rare diseases is difficult, and training dose prediction and conversion models tailored for such diseases is also challenging. Therefore, the generalizability of the DL‐based dose conversion model may be biased, and it may not necessarily demonstrate high performance for all tumor sites.

In this study, we aimed to develop a DL‐based dose conversion model that converts a PB dose into an MC dose and can be applied to any tumor site. The generalizability of the proposed model was enhanced by training on four representative tumor sites with different heterogeneities, that is, the H&N, lung, liver, and prostate, using various beam configurations. Furthermore, we investigated the model's generalizability by evaluating the conversion accuracy on testing datasets for various untrained tumor sites, including rare tumor sites where data collection is difficult.

## 2 METHODS AND MATERIALS

**FIGURE 1:**
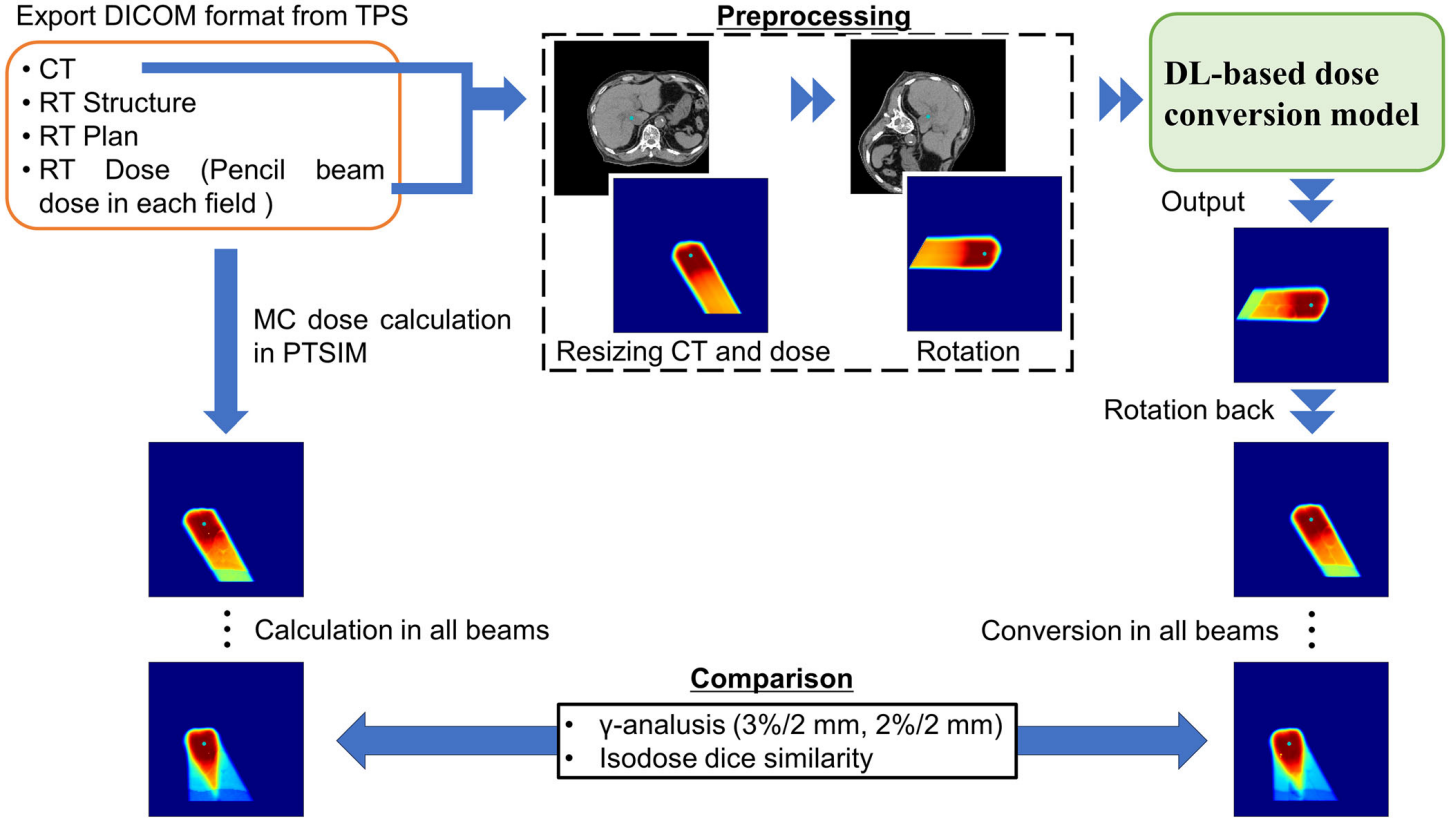
Overall workflow of the study. The cyan point indicates the isocenter. The green rotation arrow indicates the direction of rotation.

The methodology employed in this study is shown in Figure 1. First, the planning computed tomography (CT) images, structure set, plan, and PB dose distribution for each field were exported in Digital Imaging and Communications in Medicine (DICOM) format from the Xio‐M TPS (Hitachi, Kashiwa, Japan). Based on the treatment planning data, the MC dose calculations were performed using the in‐house platform, as described in the section 2.2. The CT and PB doses were then input to a DL‐based dose conversion model and converted to an MC‐equivalent dose. To enhance conversion accuracy, an image‐rotation technique was employed (explained in Section 2.3). Finally, the ground‐truth MC dose and the converted dose were compared to evaluate the performance of the model.

### 2.1 Patient characteristics

We trained a DL‐based dose conversion model using four tumor sites with different heterogeneities (H&N, lung, liver, and prostate) to develop a model applicable to any tumor site. We obtained treatment planning data for 86 patients (110 plans/362 beams) with H&N cancers, 78 patients (90 plans/192 beams) with lung cancers, 78 patients (95 plans/237 beams) with liver cancers, and 97 patients (97 plans/356 beams) with prostate cancers (Table 1). The institutional review board at Southern Tohoku Proton Therapy Center approved the use of all data considered in this study (IRB No. 599). For each trained site, the treatment plan data were randomly divided into 98, 80, 85, and 87 plans for training/validation, respectively, and 12, 10, 10, and 10 plans for testing, respectively. Tumor localization in the H&N included the oral cavity, nasal cavity, nasopharynx, oropharynx, hypopharynx/larynx, parotid gland, and cervical lymph nodes. Lung cancers included the upper lobe, middle lobe, lower lobe, and hilum. Liver cancers included the right lobe, left lobe, and bile duct region. For prostate cases, either the prostate alone or the prostate and seminal vesicles were included. Note that the target could also involve surrounding lymph node metastases in all trained sites.

**TABLE 1:** Patient characteristics for each tumor site.

				Number of beams			
	Tumor site	Number of patients	Number of plans	Total	Average	Minimum	Maximum
Trained sites	Head&Neck	86	110	362	3.3	2	4
	Lung	78	90	192	2.1	1	4
	Liver	78	95	237	2.5	1	4
	Prostate	97	97	356	3.7	2	4
Untrained sites	Esophagus	6	6	13	2	2	3
	Pancreas	14	14	38	2.7	2	4
	Colorectal	9	9	30	3.3	2	4
	Brain	2	2	6	3	3	3
	Breast	3	3	14	4.7	4	6
	Cervical	2	2	4	2	2	2
	Limb	5	6	20	3.3	2	4

To investigate the generalizability of the DL‐based dose conversion model, we also tested the model's performance on untrained tumor sites, including 6 patients/6 plans/13 beams for esophagus, 14 patients/14 plans/38 beams for pancreas, 9 patients/9 plans/30 beams for colorectum, 2 patients/2 plans/6 beams for brain, 3 patients/3 plans/14 beams for breast, 2 patients/2 plans/4 beams for cervix, and 5 patients/6 plans/20 beams for limb bone and soft tissue, as shown in Table 1. Tumor localization for the esophagus included the cervical or thoracic esophagus, the pancreas included the head, body, and tail, and the colorectum included the sigmoid colon and rectum. The pediatric brain tumors included the whole ventricle. Breast tumors included the upper inner and outer quadrants and the nipple. For limb bone and soft tissue tumors, the buttocks, short bones of the lower limbs, and forearms were included.

### 2.2 MC calculation

We employed the Particle Therapy Simulation Framework (PTSIM) for the MC calculations.^21^ PTSIM is a dedicated radiation therapy application developed based on Geant4. Our center's passive scattering proton therapy system, Melthea (Hitachi, Kashiwa, Japan), was modeled in PTSIM. The nozzle consists of a wobbler magnet, a scatterer, a ridge filter, a degrader, a multileaf collimator, and a patient‐specific bolus. At our center, three initial proton beam energies of 150, 210, and 230 MeV are clinically available, and the range is adjusted using degraders. In this study, initial proton beam energies of 150 and 210 MeV and all degraders were commissioned in PTSIM, which are frequently used clinically. For the MC dose calculation commissioning, the source energy, position, and angular distribution were adjusted based on measurements. Further details of the commissioning and MC calculations can be found in our previous publication.^22^

### 2.3 DL–based dose conversion model

We employed the Hierarchically Densely Connected U‐net (HD U‐net)^23^ for the DL‐based dose conversion model, which combines U‐net^24^ and DenseNet.^25^ This architecture has previously demonstrated high performance in DL‐based dose conversion for the PBT.^16^ In the HD U‐net, each layer uses two dense convolutions with a growth rate of 8. In the encoder, four pooling layers combine the max pooling (kernel size = 2, stride = 2) and the stride convolution (kernel size = 3, stride = 2), which are then concatenated. The decoder, similar to U‐net, receives the features from each encoder layer and performs deconvolution (kernel size = 2, stride = 2). The HD U‐net was implemented in Pytorch 2.6.0 and trained on an NVIDIA RTX 6000 Ada Generation GPU with 48 GB memory. The model was trained with the Adam optimizer with the L1 loss and an initial learning rate of 0.001. If the validation loss did not improve after 20 epochs, the learning rate was reduced by 1/2. To improve the model's performance, the DL model was trained using three‐fold cross‐validation with a batch size of 24 for a maximum of 500 epochs. If the validation loss did not improve, early stopping was performed with a patience level of 30 epochs. In addition, a patch‐based technique^26^ was employed with a patch size of 96 × 96 × 96.

The proposed DL‐based dose conversion model outputs the MC‐equivalent dose from CT images and the corresponding PB doses for each field. First, the CT images and dose distributions were interpolated to a 2‐mm grid (192 × 192 × 192). To enhance the model's performance, image‐rotation preprocessing was performed,^22^ in which the images were rotated so that the beams were virtually incident from a gantry angle of 270° and a couch angle of 0°. The output from the DL model was then rotated back to the original gantry and couch angles. The Hounsfield units in the CT images were clipped to [0–3000] and normalized by their maximum values. The PB and MC doses were also normalized by the prescribed dose for each beam.

### 2.4 Generalizability of the DL model

To evaluate the generalizability of the DL‐based dose conversion model, we developed five models, where one model was trained with four tumor sites (the generalized model), and four individual models were trained with each tumor site (site‐specific models). For the generalized model, pairs of CT images and PB doses for each beam for four tumor sites with different inhomogeneities were employed simultaneously for training and validation. For the site‐specific model, training and validation were performed for each tumor site, and four models were trained separately (i.e., the H&N model, the Lung model, the Liver model, and the Prostate model). To eliminate bias due to the number of datasets per tumor site, zooming augmentation was performed for the datasets in each tumor site.^22^ Table 2 shows the number of training and validation datasets for each tumor site model.

**TABLE 2:** NTumber of training datasets for each tumor site.

	Head&Neck	Lung	Liver	Prostate
Number of training datasets	362	192	237	356
Scaling factor	× 3	× 6	× 5	× 3
Total number of training datasets	1086	1152	1185	1068

### 2.5 Evaluation of conversion accuracy

To evaluate the conversion accuracy, 3D **γ‐**analysis (threshold 10%) was performed on the dose distribution accumulated across all fields in the testing datasets. The distance‐to‐agreement criterion was set to 2 mm, which is the same as the calculation grid size. In addition, 3% and 2% were set as the criteria for the dose difference. Schuemann et al. reported that the dosimetric errors between PB and MC doses were as much as 5% in the target.^6^ Therefore, in the present study, we set these dose‐difference criteria to strictly evaluate the degree to which the PB dose errors were improved.

In addition, to evaluate the structural accuracy of the dose distribution, we calculated the Dice similarity coefficient (DSC) for some isodose volumes. The DSC was calculated between 0% and 100% at 20% intervals relative to the prescribed dose, using the following formula:

$$DSC=\frac{2\left(V_{1}\cap V_{2}\right)}{V_{1}+V_{2}},$$

where $V_{1}$ and $V_{2}$ denote the isodose volume at the MC dose, and the PB dose or DL‐based conversion dose, respectively.

## 3 RESULTS

### 3.1 Example of dose conversion accuracy

**FIGURE 2:**
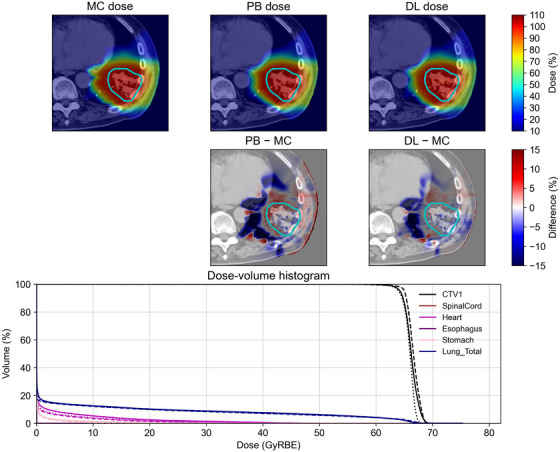
A representative example of a trained tumor site in the lung. All dose distributions were normalized to the prescribed dose. The prescribed dose was 66 Gy(RBE) in 10 fractions. The cyan lines in the top two rows indicate the clinical target volume. In the dose‐volume histogram, the solid, dashed, and dotted lines represent the MC dose, PB dose, and DL dose, respectively.

**FIGURE 3:**
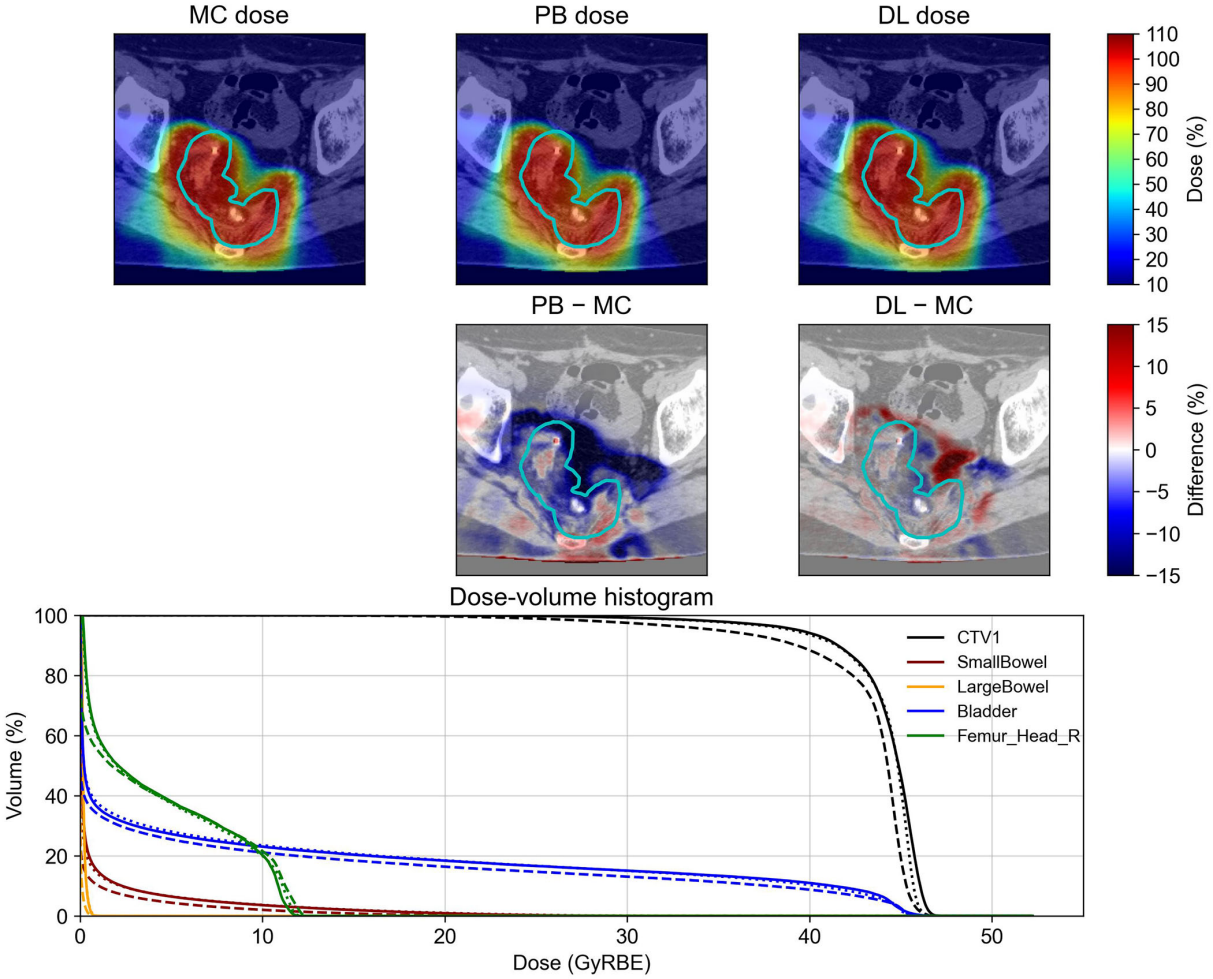
A representative example of an untrained tumor site in the colorectum. All dose distributions were normalized to the prescribed dose. This patient had a two‐step treatment plan, and these dose distributions were the first half of the plan. The prescribed dose was 45 Gy(RBE) in 15 fractions. The cyan lines in the top two rows indicate the clinical target volume. In the dose‐volume histogram, the solid, dashed, and dotted lines represent the MC dose, PB dose, and DL dose, respectively.

**FIGURE 4:**
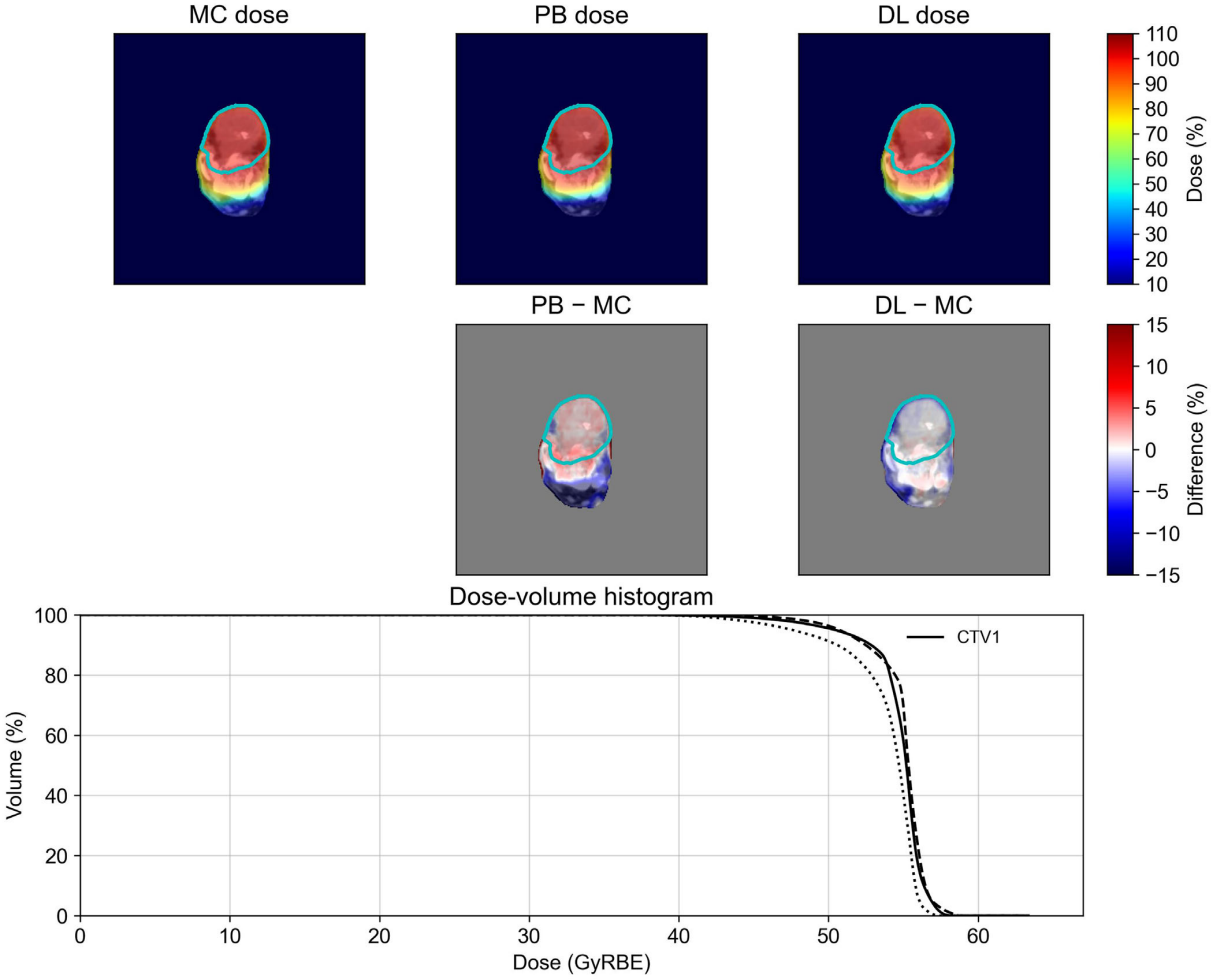
A representative example of an untrained tumor site in the limb bone and soft tissue. All dose distributions were normalized to the prescribed dose. The prescribed dose was 55 Gy(RBE) in 25 fractions. The cyan lines in the top two rows indicate the clinical target volume. In the dose‐volume histogram, the solid, dashed, and dotted lines represent the MC dose, PB dose, and DL dose, respectively.

The conversion accuracy was evaluated for both trained and untrained tumor sites. Representative examples of some tumor sites, such as the lung, colorectum, and limb bone and soft tissue, are shown in Figure 2, 3, 4, including the MC dose, PB dose, DL dose (generalized model), and corresponding dose‐volume histogram. Visual inspection demonstrated that the DL doses were more consistent with the MC doses than the PB doses. Although the DL dose showed inferior agreement compared with the PB dose in the dose‐volume histograms for the limb bone and soft tissue, the DL doses were generally closer to the MC doses than the PB doses.

### 3.2 3D γ‐analysis

**FIGURE 5:**
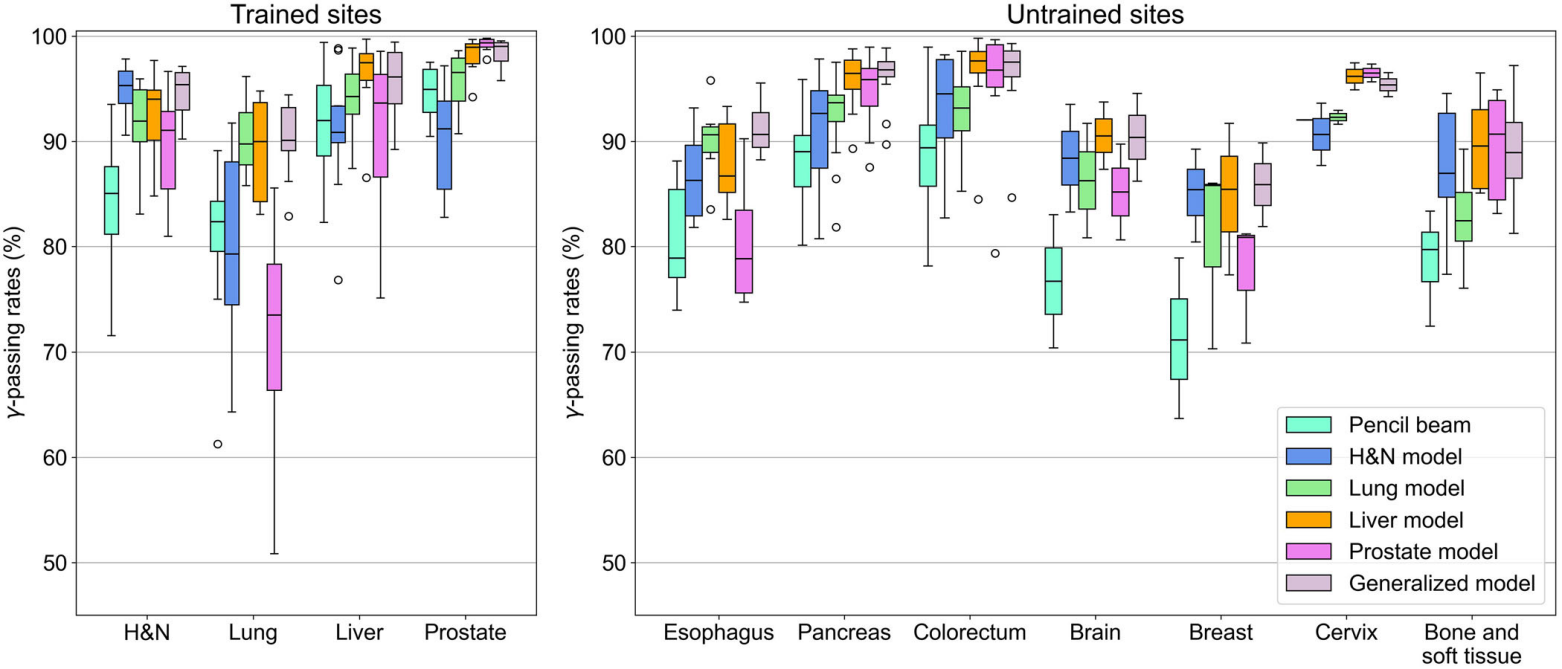
3D γ‐analysis (3%/2 mm) for trained and untrained tumor sites.

To evaluate the performance of the DL‐based dose conversion model, 3D **γ‐**analysis was performed using the 3%/2 mm and 2%/2 mm criteria. Figure 5 shows the results of 3D **γ‐**analysis with the 3%/2 mm criterion for the trained tumor sites. The generalized model indicated average γ‐passing rates of ≥90% for all trained tumor sites. In addition, the site‐specific models, which were the same as the testing tumor site, demonstrated high γ‐passing rates. However, the average passing rates for the lung cases were 85.1% for the H&N model and 77.5% for the Prostate model. Furthermore, the H&N model exhibited a relatively low average passing rate of 92.1% in the prostate cases.

For the untrained sites, the generalized model achieved the best performance, with passing rates of ≥90% for most sites. For the esophagus cases, which have heterogeneity similar to that of the lung cases, the generalized model achieved relatively high passing rates compared with the other models. The generalized, Liver, and Prostate models also performed well for the pancreas and colorectal cases, as these had homogeneity similar to the liver and prostate cases. The γ‐passing rates for the brain, breast, cervix, and limb bone and soft tissue tended to decrease overall compared with the other tumor sites. Among the results, the generalized model and site‐specific models with similar heterogeneity demonstrated relatively high passing rates.

**FIGURE 6:**
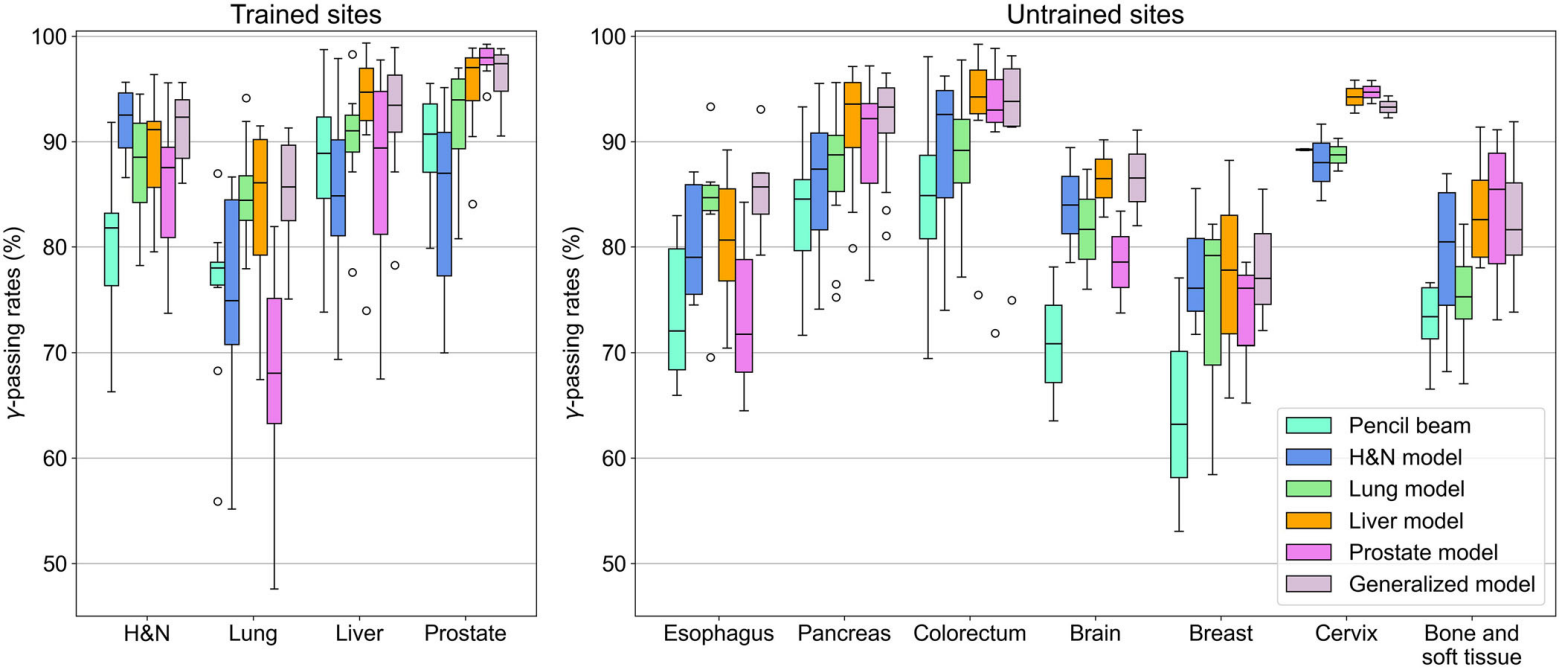
3D γ‐analysis (2%/2 mm) for trained and untrained tumor sites.

The results for the 2%/2 mm criterion showed remarkable heterogeneity effects between the testing and training sets (Figure 6); however, the generalized model consistently demonstrated superior overall performance.

### 3.3 DSC

**FIGURE 7:**
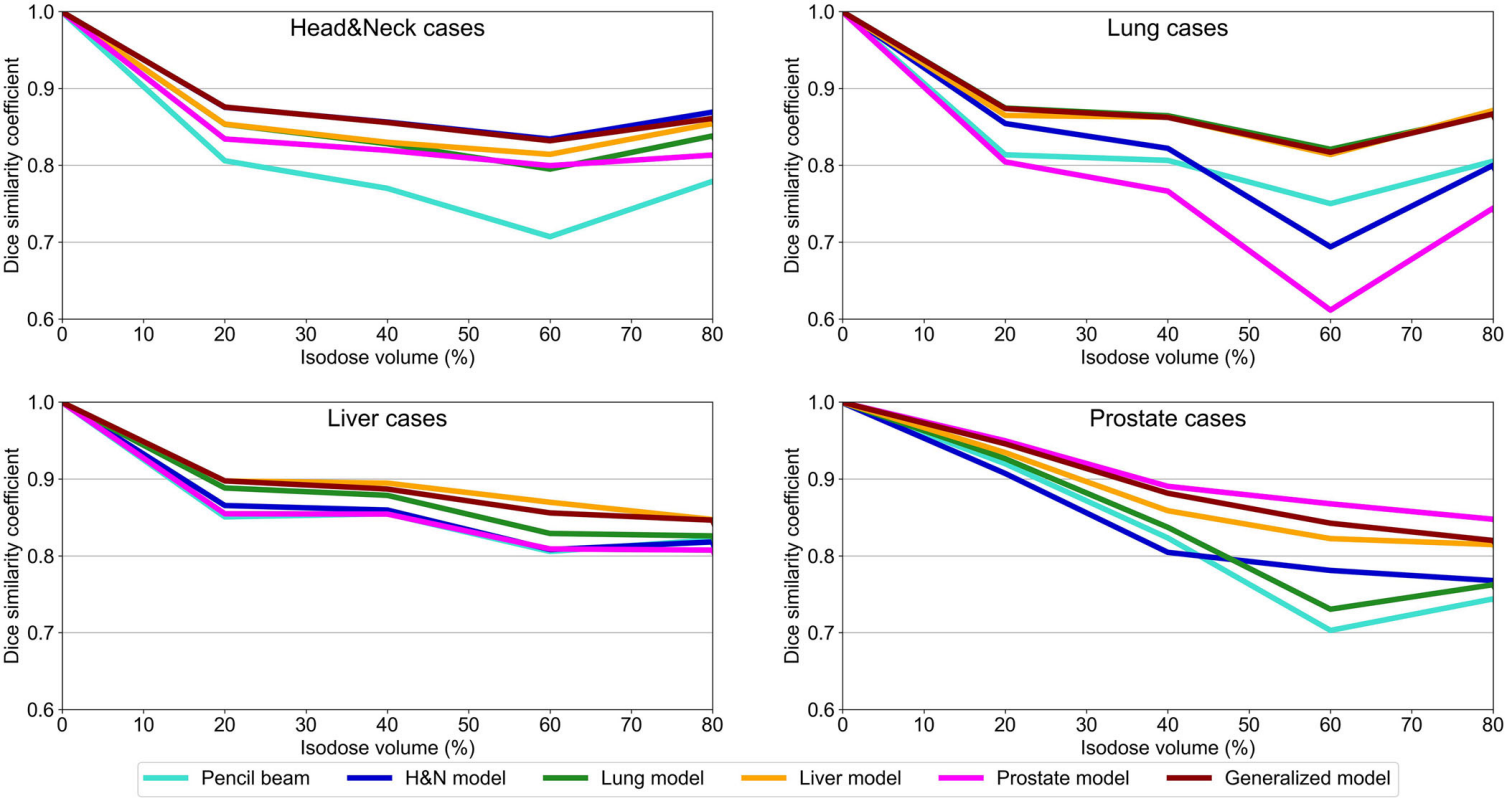
DSC in trained tumor sites.

To evaluate the geometric consistency of the dose distribution, the DSC was calculated in the 0%–100% isodose volumes. Figure 7 shows the average DSC values for each model in the testing cases of the trained tumor sites. The generalized model showed high DSC values, that is, ≥0.8 overall. In addition, the site‐specific models of the same tumor sites also demonstrated high DSC values. The DSC for lung cases tended to be low across all models, with the Prostate model showing the worst DSC values in the 60%–80% isodose volume, approximately 0.6. In contrast, the generalized and Lung models obtained high DSC values of ≥0.8, even for lung cases. For the liver cases, the generalized model and the site‐specific models also showed DSC values of ≥0.8.

**FIGURE 8:**
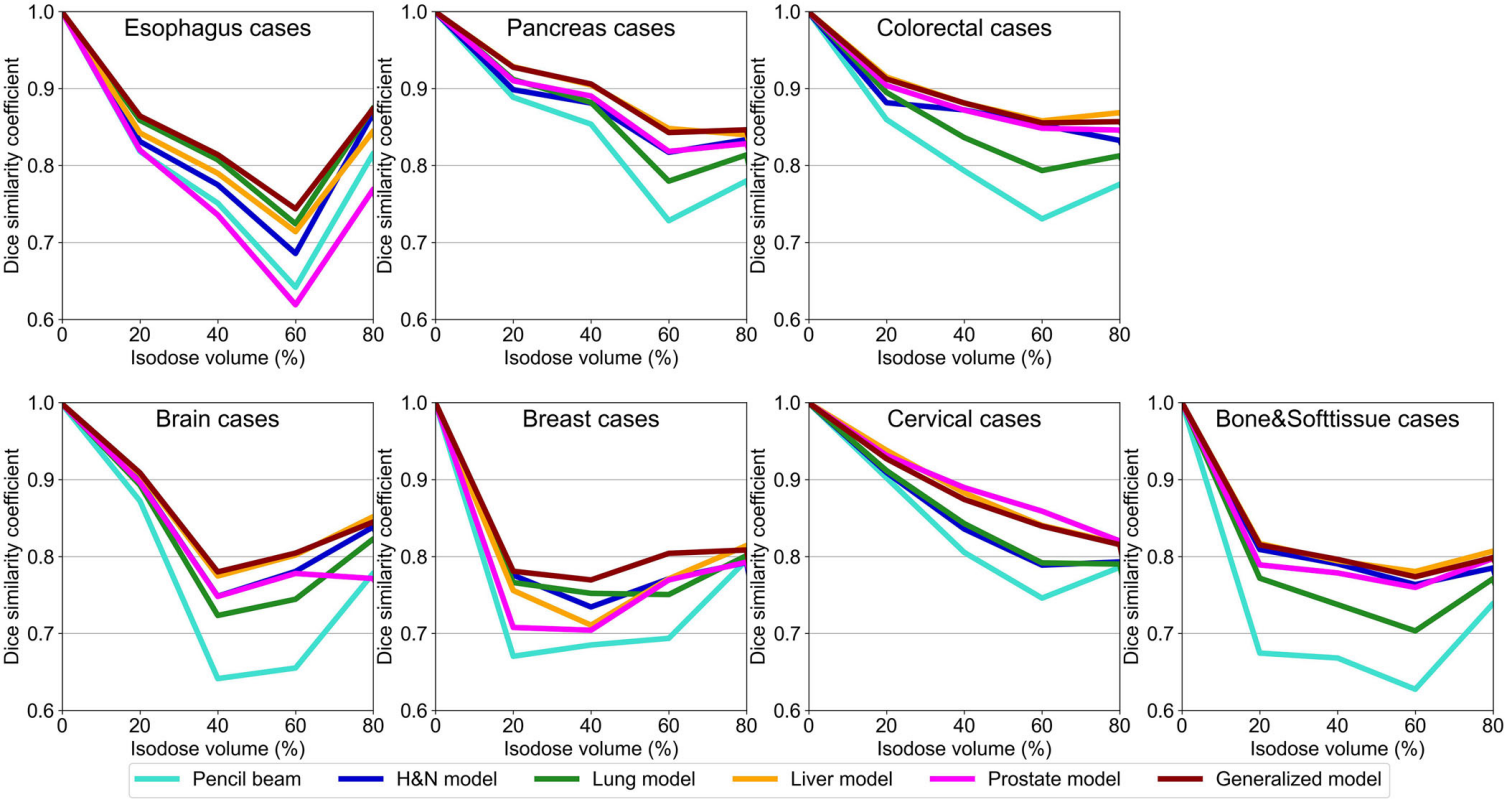
DSC in untrained tumor sites.

Figure 8 shows the average DSC values for each model in the testing cases of the untrained tumor sites. For these untrained sites, the generalized model or site‐specific models with similar heterogeneity between the testing and training sites achieved relatively high DSC values, for example, the esophagus cases in the Lung model. For the relatively homogeneous pancreatic and colorectal cases, the generalized model and site‐specific models (except the Lung model) exhibited DSC values of ≥0.8. However, for the esophagus cases, the DSC value in the 60%–80% isodose volume, even for the generalized model, was approximately 0.75. Although all models showed lower DSC values in the esophagus, brain, chest, and limb bone and soft tissue compared with other tumor sites, the generalized model demonstrated the highest DSC value of ≥0.75 in all untrained tumor sites.

## 4 DISCUSSION

In this study, we developed a DL‐based dose conversion model for PBT that can be applied to any tumor site and demonstrated its generalizability. The proposed DL‐based dose conversion model is particularly useful for TPSs, in which the MC dose calculation cannot be used, as it can rapidly generate MC‐equivalent dose distributions, thereby enabling rapid secondary checks. When the conversion accuracy is uncertain, expert review or validation using gold‐standard MC calculations should be required. Furthermore, the DL‐based dose conversion model could be useful for highly accurate retrospective reviews of patients previously treated with passive scattering PBT. By leveraging the large amount of treatment planning data from passive scattering PBT with long‐term follow‐up results, this approach may help to reconstruct the reliable evidence for PBT. This method also enables the rapid update of NTCP models previously created using the old algorithm, such as the PB, to MC‐based models.^26^, ^27^, ^28^ However, the variation and number of diseases that can be collected at each PBT center are limited, making it difficult to obtain a substantial amount of treatment planning data for all tumor sites. Therefore, it is important to demonstrate the model's generalizability, which has not yet been fully clarified. Wu et al. reported the best performance of DL‐based dose conversion trained on over 500 tumor sites, including the H&N, lung, liver, and prostate in PBT.^16^ However, they did not evaluate the model performance for untrained tumor sites. While there are several reports on DL‐based dose prediction, studies on DL‐based dose conversion models remain limited. We evaluated the model performance on untrained tumor sites, including the esophagus, pancreas, colorectum, brain, breast, cervix, and limb bone and soft tissue in the extremities, in addition to the trained tumor sites. The proposed model achieved high γ‐passing rates and DSC values even in the untrained tumor sites, thereby demonstrating its generalizability. In other words, a DL‐based dose conversion model will perform well for any tumor site if trained on datasets encompassing diverse heterogeneities. These findings suggest that the approach can be adapted to biases in collecting disease data at each PBT center and for rare diseases in PBT.

In the proposed DL‐based dose conversion model, the generalized model, which was trained on various tumor sites with different heterogeneities, demonstrated the best overall performance. However, compared with the results reported by Wu et al.^16^ and studies on other dose distribution predictions and calculations using DL in PBT,^17^, ^18^, ^19^, ^20^, ^29^, ^30^, ^31^ the γ‐passing rate in our results tended to be a little lower. We believe that these discrepancies are largely due to the diversity of the training data. Our model was trained on multiple tumor sites, as described in the Methods section, using PB doses in clinical treatment plans and MC doses recalculated in PTSIM. Note that these doses vary significantly among individual patients, depending on different factors, such as tumor location, the number of targets, and beam configurations. Nevertheless, our primary goal is to demonstrate the generalizability of the proposed DL‐based dose conversion model. We found that the generalized model, which was trained on four representative tumor sites with different heterogeneities, also performed well on untrained sites. These results suggest that training a model on diverse datasets with different heterogeneities can be applied to any tumor site. In addition, when the heterogeneity between the testing and training datasets was similar, the site‐specific model also performed relatively well. These results provide valuable information for cases where limited training data are available.

The proposed DL‐based conversion model demonstrated high performance for any tumor site; however, the conversion accuracy for some untrained sites was slightly lower than that of other sites. We believe that this was due to the discrepancies in heterogeneity and the size of the object. To further improve the accuracy, DL techniques such as transfer learning and fine‐tuning processes may be effective. Previous studies have investigated the dose distribution prediction tasks by adapting models to different tumor sites and employing treatment techniques that utilize transfer learning and fine‐tuning.^32^, ^33^, ^34^ For instance, Mashayekhi et al. adapted a dose distribution prediction model for intensity‐modulated radiation therapy in the prostate to volumetric modulated arc therapy in the H&N using transfer learning.^34^ In addition, Moore et al. improved the accuracy of a dose prediction model in cervical cancer brachytherapy by customizing it for each device using a dataset with various tools, for example, applicators and needles.^35^ In the dose conversion task, we found that training a model for multiple tumor sites rather than specific sites improves applicability to heterogeneity and facilitates the development of more versatile models. These techniques can be effective to further improve conversion accuracy, even for rare tumor sites where data collection is challenging. Furthermore, designing the model is desirable at multiple institutions to train on more tumor sites and treatment planning data in the future.

This study has several limitations that should be acknowledged. First, the evaluation of heterogeneity was qualitative, and it was not possible to quantify the heterogeneity at each tumor site. However, if heterogeneity at each tumor site can be quantified using a reliable method, it could potentially be effective in identifying tumor sites where the site‐specific models are applicable and in developing more universal DL‐based dose conversion models. Second, there was a bias in the evaluation of the untrained regions. Due to the differences in the diseases that can be collected at each PBT center, the analysis performed in the current study considered only a small number of cases, for example, pediatric, cervical, and breast tumors. Increasing the number of cases for these tumor sites may yield more general results. However, the goal of this study was to investigate whether a model trained on representative tumor sites in PBT can be applied to untrained sites. The findings of this study have demonstrated that if a large number of datasets with different heterogeneities can be gathered, the model can achieve reasonably high performance even for untrained sites. Finally, this study was conducted using a passive scattering system, and the proposed model has not been implemented on a PBS system. The proposed DL‐based dose conversion model is useful for improving the accuracy of treatment planning at PBT centers where MC dose calculations cannot be performed using TPS, especially in passive scattering systems. Although the performance of the DL‐based dose conversion model on PBS systems is unknown, we believe that the proposed model is sufficiently compatible with PBS systems. Wu et al. demonstrated that a model trained on datasets in a passive scattering system could be adapted to intensity‐modulated proton therapy using transfer learning.^16^ Thus, if our model introduced such a DL technique, it should also be applied to PBS systems. Furthermore, while calculation time was beyond the scope of this study, our previous research demonstrated that the DL‐based dose conversion model could calculate the MC‐equivalent doses in less than 1 s,^22^ and the calculation times observed in the current study were comparable. Therefore, if applicable to PBS systems, the proposed DL‐based dose conversion model may prove highly beneficial in online adaptive PBT, which requires fast dose calculations. Furthermore, the DL‐based dose conversion model could potentially be expanded to convert dose distribution into linear energy transfer maps, enabling biologically based dose evaluations.^36^

## 5 CONCLUSION

In this study, we investigated the generalizability of a DL‐based dose conversion model trained on four representative tumor sites with different heterogeneities in PBT. For various tumor sites, the generalized model and site‐specific models with similar heterogeneity demonstrated high performance. The results demonstrated the ability of the proposed model to calculate dose distributions comparable to MC for any tumor site using the generalized model. In addition, the site‐specific models are likely to achieve relatively high conversion accuracy if the heterogeneity is comparable. These findings suggest that the proposed method can be adapted to biases in disease data collection at each PBT center and for rare diseases in PBT.

## AUTHOR CONTRIBUTIONS

RK, NK, and TK contributed to the conception and design of this study. RK and SO performed data collection. RK, RT, and SO conducted the primary analysis. RK mainly drafted the manuscript. NK, TK, MM, and KJ reviewed the manuscript. All authors read and approved the final manuscript.

## CONFLICT OF INTEREST STATEMENT

The authors declare that there are no conflicts of interest.

## ETHICS STATEMENT

This study was approved by the institutional review board at Southern Tohoku Proton Therapy Center (IRB No. 599).
